# Fidelity in Archaeal Information Processing

**DOI:** 10.1155/2010/960298

**Published:** 2010-09-05

**Authors:** Bart de Koning, Fabian Blombach, Stan J. J. Brouns, John van der Oost

**Affiliations:** Laboratory of Microbiology, Wageningen University, Dreijenplein 10, 6703 HB Wageningen, The Netherlands

## Abstract

A key element during the flow of genetic information in living systems is fidelity. The accuracy of DNA replication influences the genome size as well as the rate of genome evolution. The large amount of energy invested in gene expression implies that fidelity plays a major role in fitness. On the other hand, an increase in fidelity generally coincides with a decrease in velocity. Hence, an important determinant of the evolution of life has been the establishment of a delicate balance between fidelity and variability. This paper reviews the current knowledge on quality control in archaeal information processing. While the majority of these processes are homologous in Archaea, Bacteria, and Eukaryotes, examples are provided of nonorthologous factors and processes operating in the archaeal domain. In some instances, evidence for the existence of certain fidelity mechanisms has been provided, but the factors involved still remain to be identified.

## 1. Introduction

Francis Crick first announced his central dogma of molecular biology in 1958: the flow of sequential information that occurs in living cells, including replication of stored information (DNA), as well as expression of this information via messengers (mRNA) to functional proteins [1]. This dogma turned out to be a solid basis for molecular biology, although additional roles of (small) regulatory and metabolic RNA have been recognized more recently [2]. A key element during this transfer of genetic information is fidelity: the final accuracy depends on the combined error rates of the processes that constitute the whole chain. 

From the ancient RNA world on, replication fidelity has been a major limiting factor of the amount of information stored. It has been proposed that on average less than one error per replicated genome is tolerated, as higher error rates lead to a so-called “error catastrophe” with a fatal amount of progeny not being viable [3–5]. The same rule applies also for extant cellular life in which double-stranded DNA is used for storage of genetic information. The increase in genome size was allowed by the increased stability of DNA [6] and by considerably lower error rates in DNA replication [7]. One might expect a continuous selection towards the highest possible fidelity. However, a very high level of fidelity in replication will negatively affect both the genome's adaptation potential, and the replication velocity and costs, posing the risk of being out-competed by more efficient rival organisms [8, 9]. Overall, the delicate balance between fidelity and mutation rate is in itself a trait of organisms and can differ between individuals and species [10]. For some species it is even known to change upon environmental signals and may vary between different locations within the same genome [11]. Fidelity of information processing is thus a major factor driving the evolution of cellular life. 

Transcription and translation show significantly higher error rates than replication. Although the risk on affecting progeny is lower, erroneous gene expression might influence the error rate of replication indirectly, for example, when the replication machinery is affected [12]. On the one hand, inaccurate gene expression may lead to the production of nonfunctional proteins, and as such to a decreased fitness, that is, generating selective pressure for increasing fidelity. On the other hand, increasing the fidelity of transcription and translation also correlates with decreasing velocity, what also has an impact on fitness. Hence, natural evolution leaves a narrow range for varying the level of fidelity [13, 14]. 

In this paper, we will, whenever possible, focus on the systems of Archaea that contribute to accurate replication and expression of their genetic information. While the majority of the archaeal processes are well conserved in Bacteria and/or Eukaryotes, a number of examples will be described of factors and processes that appear to be restricted to the archaeal domain. Despite the fact that research on Archaea is generally lagging behind that of the other two domains, the successful development of several Archaea as model organisms has recently lead to some first insight in their mechanisms to control fidelity of information processing.

## 2. Replication

Fidelity in replication is the result of three separate processes: (i) base selection, (ii) proofreading, and (iii) postsynthetic correction [15, 16]. These three processes contribute to very accurate DNA replication: incorporating a mistake only once every 10^6^–10^10^ nucleotides for DNA-based microorganisms. Interestingly, the genomic mutation rate (the number of mutations per replicated genome) is quite constant for all DNA-based microorganisms, including bacteriophages, bacteria, and fungi: roughly 0.003-0.004 (Drake's rule [7]), what is largely below the above mentioned predicted upper limit of 1 error per replicated genome [4]. Surprisingly, it has recently been found that a thermophilic bacterium (*Thermus thermophilus*) and a thermophilic archaeum (*Sulfolobus acidocaldarius*) have error rates that are 5-fold lower, supporting the concept that there is an evolved balance between the need for fidelity and the cost of reducing the mutation rate [17].

After a brief description of polymerases in living systems, the three separate processes will be discussed in more detail. The last paragraph will discuss systems that organisms have evolved to overcome misincorporations.

### 2.1. DNA Polymerases

DNA is polymerized by DNA-dependent DNA polymerases (DNAPs) that can be classified into various families based upon their sequence similarity. Most replication-related DNAPs and primases belong to DNAP family B. Like Bacteria and Eukaryotes, Archaea contain multiple DNAPs. *Sulfolobus solfataricus*, for example, contains three family B DNAPs (B1 to B3) and one family Y DNAP (Dpo4) [18]. Crenarchaeota are restricted to family B for their replicative polymerases, while Euryarchaeota, Korarchaeota, Nanoarchaeota, and Thaumarchaeota use both a family B and a family D DNAP [19]. There is biochemical evidence that in these species the family B DNAP replicates the leading strand, while the family D DNAP replicates the lagging strand [20]. Deviation between leading and lagging strand replication has been found in other domains of life as well [21, 22]. Lagging strand replication involves Okazaki fragments that are produced by a lagging strand replicative DNAP, initially extending an RNA primer generated by a primase, a family B RNA polymerase. Archaea possess homologs of eukaryotic primase proteins (PriS and PriL) that can synthesize both RNA as DNA oligonucleotides in vitro, but seem to prefer RNA polymerization in vivo [23, 24]. Interestingly the B family replicative DNAPs of Archaea contain an uracil-specific pocket that scans the template for the presence of uracil ahead of the polymerase. This feature is apparently lost in eukaryotic and bacterial DNAPs, although they still possess the reminiscent pocket structure. If uracil is encountered the archaeal polymerase stalls, presumably until the uracil is removed by Base Excision Repair (BER) or until a Translesion Synthesis (TLS) DNAP takes over [25, 26]. TLS is a process in which the regular replicative DNAP is substituted by a translesion DNAP. Translesion polymerases, often family Y DNAPs, allow replication to occur past otherwise impassable DNA lesions. This adaptation however has led to a considerably lower fidelity than in case of replicative DNAPs. Dpo4 from *Sulfolobus solfataricus* is a family Y TLS DNAP. Dpo4 has a spacious solvent-exposed active site in comparison to replicative DNAPs that permits accurate bypass of the 8-oxoguanine oxidation product of guanine. 8-oxoguanine preferentially base-pairs to adenine, however in the active site a stabilizing hydrogen bond network fixes 8-oxoguanine in such position that the correct preference for cytosine is restored [15, 27].

### 2.2. Base Selection

The highest contribution to fidelity during DNA replication is brought about by base selection. Soon after the initial suggestion by Watson and Crick that selection was the result of hydrogen bonding of complementary bases [28], it became clear that the free-energy differences between correct and incorrect base-pairs could only account for error rates of approximately 0.01 [16]. Although the removal of water from the active site of the DNAP leads to elevated ΔG values, improving the selectivity between correct and incorrect base pairings [29], studies with base analogs that lost the capacity to create hydrogen bonds revealed the importance of base pair geometry. In addition, structural studies showed that a Watson-Crick pair, of which all four are nearly identical in shape and size, fits nicely into the base pair binding pocket of DNAP, while non-Watson-Crick base pairs presumably cause steric clashes (reviewed in [15, 30]).

### 2.3. Proofreading

Like the replicative DNAPs of the Bacteria and Eukaryotes, both family B as family D DNAPs from Archaea possess intrinsic proofreading capabilities [26, 31, 32]. Because these enzymes are thermostable, and have intrinsic proofreading, they are of commercialy interest as exemplified by the high-fidelity Pfu DNAP from *Pyrococcus furiosus* in polymerase chain reactions. Comparisons between wild-type polymerases with intrinsic proofreading capabilities and exonuclease-deficient mutants show that on average proofreading improves fidelity between 3–100 fold. For *Sulfolobus solfataricus* DNAP B1, the commercially available DNAP (Vent pol) from* Thermococcus litoralis* and their respective exonuclease-deficient mutants, it was measured to improve approximately 3 fold, a similar increase as observed for* E. coli* DNA pol III [15, 32].

DNAPs have prolonged interaction with the newly generated duplex DNA. Mismatches are recognized because of abnormal base pair geometry, and generally result in considerably decreased elongation rate. In DNAPs that have intrinsic or associated 3′→5′ exonuclease activity, elongation rate drops below the exonuclease rate upon mismatch recognition, leading to removal of mismatched nucleotides. Polymerases without intrinsic exonuclease activity can either recruit another protein that has exonuclease activity, or can dissociate and allow another polymerase with intrinsic exonuclease activity to take over. 

 Other errors generated during elongation, at approximately the same rate as mismatches, are single-base deletions and slightly less frequently single-base insertions. These “indels” can occur by (i) DNA strand slippage, (ii) misinsertion that is followed by primer relocation, or (iii) misalignment at the polymerase active site, and can occur especially at repetitive sequences. Whereas proofreading corrects mismatches at a high rate, this mechanism is relatively inefficient in correcting indels, especially if the repetitive elements are longer. Strand slippage, for example, occurs often upstream of the polymerase, is therefore not sensed and does not decrease the elongation rate, preventing the exonuclease activity from taking over (reviewed in [15, 30]).

### 2.4. Postsynthetic Correction

Mismatches or indels that slipped through the proofreading process, or that are introduced by mutagenic factors, are to be repaired by postsynthetic correction. Organisms generally have a set of distinct systems, designed to repair a specific class of damage, each with a different fidelity rate. A repair system directly connected to replication is Mismatch Repair (MMR). This system removes base substitutions and indels on the newly synthesized strand directly after replication. MMR increases fidelity of replication almost 100-fold [15]. In Bacteria and Eukaryotes, essential proteins required for MMR belong to the MutS and MutL family. These two families are largely absent in the archaeal domain. Archaeal homologs have only been found in some euryarchaeal species, probably the result of a horizontal gene transfer from bacterial origin [33]. Deletion mutants of a variety of MutS and MutL homologs in *Halobacterium salinarum,* including a MutS double mutant, had only little effect on mutation rates, indicating that these genes are not essential for MMR in this species [34]. A MutS2 ortholog is also present in the euryarchaeote *Pyrococcus furiosus* and it was shown to have ATPase and DNA binding activity, but no specific MMR activity [35]. Despite the general absence of MutS and MutL in Archaea, it is found that spontaneous base pair substitution rates in *S. acidocaldarius* are an order of a magnitude lower than MMR-proficient *E. coli* suggesting the existence of a powerful, yet unknown MMR system in Archaea [17].

 During MMR, a key step is to identify which of the two strands is the (correct) parental strand and which one the (mutated) daughter. In some bacterial systems, the methylated strand is considered to be the parental strand, a signal for MutH to cleave opposite of a methylated GATC sequence near the mismatch [36]. Other Bacteria, Eukaryotes, and Archaea use other mechanisms to distinguish between the strands that are not yet fully understood. It is believed that in Eukaryotes the newly synthesized daughter strand contains discontinuities, caused by the separate Okazaki fragments during lagging strand replication and by reinitiation or low-level incorporation of dUMP during leading strand replication. Archaea may also use the incorporation of uracils as a marker for the daughter strand as well, in line with the fact that DNA replication in Archaea cannot pass uracils on the template strand [37].

### 2.5. Excision Repair

Two additional repair systems that repair single strand damage by using the complementary strand as a template include (i) Base Excision Repair (BER) used to remove regularly occurring small, nonhelix-distorting base lesions (e.g., modification by depurinations and deaminations) and involves DNA glycosylases, and (ii) Nucleotide Excision Repair (NER) used to remove bulky distortions in the helix (e.g., thymine dimers formed by oxidative stress or UV). The BER system appears to be functional in Archaea, as archaeal BER-related thermostable N-glycosylases have been characterized [38–42]. In contrast, the archaeal NER system appears to lack important damage-recognition proteins, but has structure-specific nucleases, homologous to eukaryotic NER nucleases [37]. UV stress experiments with *Sulfolobus acidocaldarius* show evidence for the existence of an archaeal NER system, as its repair capacity is at least half the capacity of NER-proficient *E. coli* [43, 44]. Especially life at elevated temperatures asks for efficient repair systems, as spontaneous decomposition reactions are accelerated under these conditions [6]. The high temperatures characterising the habitat of *Sulfolobus* species causes high rates of depurinations and deaminations. Although most of these types of damage are removed by BER, the apparent absence of key factors for both NER and MMR has been referred to as “the great irony” [37].

## 3. Transcription

During transcription mRNAs are generated by a DNA-dependent RNA polymerase (RNAP). The polymerization reactions of RNA and DNA show several similarities, for example, the course of nucleic acids through the active centre, and the mechanism of substrate binding, as reflected by the similar location of the two metal binding sites in the active sites of both polymerases [45]. Despite these similarities there are also several differences: (i) RNA polymerization incorporates NTPs instead of dNTPs, (ii) most RNAPs, with the exception of bacteriophage and mitochondrial RNAPs, are complexes that consist of 5–15 polypeptide subunits, in contrast to most DNAPs and primases that contain a single or only a few subunits, and (iii) while in DNAPs the newly formed DNA duplex persists, the newly formed RNA is removed from the DNA-RNA hybrid in RNAPs after which the original DNA duplex is restored [45].

 Two processes are relevant in terms of transcription fidelity: base selection, and proofreading; post-synthetic correction of RNA does not exist, although some systems exist to monitor the quality of the transcripts that are used as templates during translation. These surveillance systems occur mainly during translation and will be discussed in that section (later). Although the fidelity of the transcription process is considerably lower than that of the replication process, it has been reported to be less than one error every 10^5^ nucleotides that are being transcribed in organisms ranging from *E. coli *to wheat [46–48].

### 3.1. RNA Polymerases

The RNAP of Bacteria is a relatively simple complex consisting of 5 subunits. In addition, a set of up to 20 sigma factors allows for promoter selection in response to changing conditions. Eukaryotes use up to five variant RNAP complexes (I–V) that are responsible for transcription of distinct genes: ribosomal RNAs (RNAP I), protein-coding messenger RNAs (RNAP II), transfer RNAs, and other small noncoding RNAs (RNAP III). RNAP IV and RNAP V are restricted to plants and transcribe small RNAs involved in silencing [49]. RNAP I and III are similar to RNAP II, but have some additional subunits that vary between the two. Archaea, in contrast, have only a single RNAP complex that contains 12 orthologous subunits of the eukaryotic RNAP II. There appear to be minor variations among the complexes of the archaeal phyla [50, 51]. For instance, the RNAP from *Sulfolobus shibatae* has an additional subunit in comparison to the eukaryotic RNAP II (Rpo13) that has been proposed to play a role in the formation of the transcription bubble [52]. The subunits of these RNA polymerases can be assigned to three different functional groups: (i) the “catalytic core” (the large subunits A′A′′, and B′B′′; in some Archaea these subunits are fused as in Bacteria and Eukaryotes) that harbours the active site, (ii) the “assembly platform” (D, N, L, and P), and (iii) the “auxiliary subunits” (H, K, F, E, and Rpo13). The latter auxiliary set is the part of the complex that differs between the archaeal and the different eukaryotic RNAPs. These subunits that are not required for in vitro transcription, but important to stabilize interactions with RNA (F/E stalk), DNA (H and Rpo13), and transcription factors (F/E stalk). Additionally, the F/E stalk is found to be important for processivity during elongation, and correct recognition of weak terminators during termination [51, 53]. Recently it was shown that subunit H is required during promoter opening and initial transcription, and that it, in contrast to its eukaryotic counterpart Rpb5, undergoes a structural rearrangement in the transition from initiation complex to elongation complex that might be specific for archaeal RNAPs [54]. It was also shown recently that in vitro reconstitution of the archaeal RNAP is similar in the presence or absence of subunit P. Apparently it does not play a key role in establishing the assembly platform in vitro. In addition, subunit P seems to be involved in open complex formation [55]. Interestingly, a putative ortholog of Rpc34, which is a part of the eukaryotic RNAP III, has recently been found to be present in all crenarchaeal and thaumarchaeal genomes, as well as in several euryarchaeal genomes. This finding suggests that in Archaea the single RNAP might use a variable set-up of auxiliary proteins to transcribe different sets of transcripts [56]. Archaeal RNAPs can be reconstituted from single heterologously expressed subunits in contrast to eukaryotic RNAPs [57, 58]. Recent success with a hybrid archaeal enzyme that contain subunits Rpb5 and Rpb12 from Eukaryotes confirms the high structural similarity of the archaeal and the eukaryotic RNAPs [55, 59]. 

### 3.2. NTP Selection and Induced Fit

RNAPs discriminate NTPs over dNTPs by recognizing the 2′-hydroxyl group of incoming NTPs. Selection of NTPs by RNAPs is performed by measuring the base pair geometry, in a similar manner as in DNAPs, in a two step process. In the preinsertion state of the open active center the NTP can come in. If the NTP is complementary to the template nucleotide, the catalytic subunit undergoes a conformational change to the closed state, after which NTP is delivered to the insertion site. This rearranges the active site in such a way that it promotes polymerization by induced fit. If a noncomplementary nucleotide is incorporated, the complex enters an off-line state, in which elongation is slowed down considerably [60].

### 3.3. Proofreading

Incorporation of a noncomplementary nucleotide induces an inactivated state, in which the nucleotide is frayed. The fraying sites of the RNAP overlap with the NTP-binding site, and as such the frayed nucleotide does not allow elongation to proceed. This paused RNAP complex favours backtracking, a process in which the RNAPs moves one nucleotide backwards. During this process the misincorporated nucleotide is moved from the fraying site to the proofreading site. Multisubunit RNA polymerases contain an intrinsic nucleolytic RNA cleavage activity that hydrolyses a phosphodiester bond to remove the last two nucleotides as a dinucleotide, resulting in a new RNA 3′-OH group and an empty NTP-binding site. This restores an active on-line state ready for elongation again [60]. This process of backtracking and subsequent cleavage is transcriptional proofreading, and was also described in Archaea. In contrast to Bacteria and Eukaryotes, it was found that elongation in Archaea could not continue after misincorporation, but stalled completely instead. TFS, like its eukaryotic homolog TFIIS and its bacterial non-orthologous counterparts GreA/GreB, is known to induce the cleavage activity by direct interaction with the active centre of the polymerase through the nucleotide entrance pore, and could therefore rescue stalled elongation complexes. Stalling of the elongation complex in Archaea appears to be an important trigger for TFS induced cleavage in vitro [61]. *Methanopyrus kandleri* has lost TFS during its evolution. Interestingly, this organism shows a higher mutation rate in comparison with closely related organisms, making it difficult to reconstruct its phylogeny. Especially genes encoding proteins related to transcription are affected, and could include compensatory mutations for the loss of TFS [12].

## 4. Protein Synthesis

The overall missense substitution rate of in vivo bacterial protein synthesis by ribosomes is in the range of 6 × 10^−4^ to 5 × 10^−3^ per amino acid [62, 63]. In line with those findings are measurements of the rate of misreading in *Sulfolobus *in vitro translation systems: 3 × 10^−3^ incorrect leucine incorporations per amino acid on a poly(U) template [64]. Rates of misincorporations during replication, transcription, and aminoacyl-tRNA synthesis are all lower, showing that the final step, the translation process itself, is decisive with respect to fidelity of protein synthesis. The importance of fidelity during protein synthesis is reflected in the organization and evolution of the genetic code. The presumable primordial genetic code that codes for an original set of 10 amino acids [65], as well as the 20 amino acid genetic code, operating in extant cellular life forms, are relatively robust, as most misincorporations will result in substitutions by physicochemically related amino acids that only in rare occasions will lead to a nonfunctional protein [65]. Fidelity was thus a key determinant in the evolution of the genetic code. Two separate processes are distinguished during protein synthesis: the coupling of amino acids to their respective tRNAs by a set of specific aminoacyl-tRNA synthetases, and the actual translation itself by ribosomes. In the next paragraphs both processes will be discussed, after which it will be concluded with an overview of the mRNA surveillance systems that are used to avoid the reuse of erroneous templates.

### 4.1. tRNA Modification

tRNA molecules are among the most strongly modified RNAs. This mainly concerns nucleotides that are located within the 3D-core and in the anticodon arm, especially at the wobble position N34 and N37 (conventional numbering). At present, over 120 different posttranscriptional modifications of nucleotides have been described, ranging from quite simple methylations to very complex multistep transformations [66]. These nucleotide modifications are important for cellular functionality of tRNAs: they lower conformational flexibility, improve (thermal) stability, and improve aminoacylation rate and specificity. Interestingly, it is known that lack of modification in in vitro translation systems can be compensated by excess of magnesium ions, indicating the importance to lower flexibility of tRNAs for translation (reviewed in [67]). Modifications of the wobble position N34 are common in all three domains of life and contribute to accuracy and efficacy of decoding during translation. These modifications are specific and vary between tRNAs. In contrast to unsplit codon boxes in the genetic code, tRNAs coding the split codon boxes are always modified at N34, suggesting that modifications play an important role in increasing the discriminative characteristics between near-identical codons. Remarkably, many modifications of N34 are restricted to specific phylogenetic Domains, or even to lower taxonomic groups, and come with an enormous diversity. This suggests that the corresponding modification enzymes evolved after the divergence of the three domains, and that the extension of the primordial code, and the accompanying increasing need for higher discrimination capacity, has led to a multitude of solutions (reviewed in [68]). 

**Figure 2:**
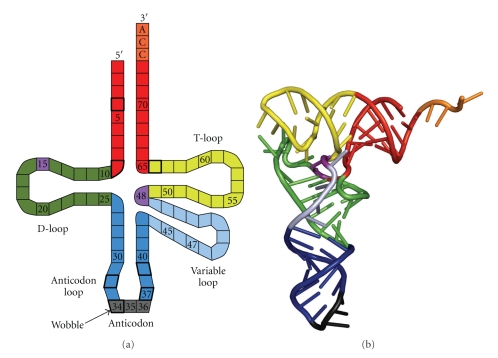
tRNAs. (a) Schematic representation, showing the D-loop (green), anticodon loop (blue) that harbours the anticodon (grey), variable loop that is variable in length (light blue), the T-loop (yellow), the acceptor stem (red), and the CCA aminoacyl binding site (orange). The Levitt base pair is coloured purple. Nucleotides with thick boxes are often modified with variable modifications. (b) Tertiary structure of a yeast tRNA^Phe^, coloured similar to (a). Figure is rendered with PyMOL from data deposited in the Protein Data Bank (1 ehz).

One of these wobble modifications in Bacteria and Eukaryotes is the conversion from G34 to queuosine. The replacement of guanine in this process is catalysed by the enzyme tRNA-guanine Transglycosylase. In Archaea, a related enzyme catalyses also the replacement step in the conversion from a guanine to a the positively charged archaeosine at position 15 [69]. G15 is part of the Levitt base-pair, which is the base-pair between N15 of the D-loop and N48 in the variable loop at the start of the T-loop, [70] and also interacts with N59 in the T-loop (Figure 2) [71]. These interactions between the D- and the T-loops establish the L-shape of the tRNA, indicating that formation of archaeosine is involved in stabilization of the RNA molecule. In Eukaryotes and Bacteria, where position 15 is not restricted to a G, and other variants of the Levitt base-pair exists, stabilization of the Levitt base-pair is brought about by Mg^2+^ binding. Interestingly, binding of a metal, which is less stable at high temperatures than chemical modification, is not compatible with archaeosine formation, suggesting distinct evolutionary mechanisms to stabilize the L-shaped structure of tRNAs between the Domains [72]. For modification of the deeply buried position 15, but probably also for other modifications, the tRNA has to adopt a different configuration, the *λ*-form. The energetics involved in such rearrangement suggest that modification enzymes might act together in a tRNA maturation complex [71]. Modifications in tRNAs are important for fidelity, processivity, and velocity of translation as they can directly affect decoding for example by modifications in the anticodon loop or in sites that are recognized by aminoacyl-tRNA synthetases, or indirectly by decreasing the flexibility and increasing the stability of the molecule.

### 4.2. Aminoacylation

The specific coupling of amino acids to their tRNAs yields aminoacyl-tRNAs (aa-tRNAs) and is catalyzed by specific aminoacyl-tRNA synthetases (aaRSs). Two classes (I and II) of aaRSs are distinguished on the basis of their structural topology of the active site [73]. Class I aaRSs, are generally monomeric, attach to the minor groove of the tRNA acceptor stem, and aminoacylate the terminal adenosine of the tRNA at the 2′-OH position, while Class II are generally multimeric, attach to the major groove, and aminoacylate the 3′-OH position [74]. Aminoacylation is a two-step process. First the amino acid is activated using ATP, forming the intermediate aminoacyl-adenylate. Once activated, the amino acid is transferred to the 3′ adenosine of the corresponding tRNA [74]. In Archaea and Eukaryotes, aaRSs are often organized in higher-order complexes that contain multiple aaRSs and other cellular factors, for example, the large multiaminoacyl-tRNA synthetase complex in *Haloarcula marismortui* that might harbour all aaRSs [75], or the LysRS-LeuRS-ProRS complex in *Methanothermobacter thermoautotrophicus* that increases the kinetics of LysRS and ProRS [76]. In Eukaryotes complex formation is sometimes also associated with other noncanonical functions like translational silencing, transcriptional control, or antiapoptosis (reviewed in [74]).

 A cell might contain over 25 different types of aa-tRNAs [77]. For translation purposes there are 20 canonical elongator tRNAs, usually acylated by the corresponding synthetases, and an initiator aa-tRNA, acylated by methionyl tRNA synthetase. In Bacteria and eukaryotic organelles, the initiator Met-tRNA^Met^ is subsequently formylated by a specific formyltransferase, in contrast to the situation in Eukaryotes and Archaea. In addition, a small number of noncanonical elongator tRNAs have been discovered (selenocysteinyl-tRNA, and pyrrolysyl-tRNA; see below). After coupling, aa-tRNAs are screened for their correctness by the translation elongation factor EF-Tu (eEF1A/aEF1*α* in Eukaryotes and Archaea) and delivered to the ribosome, with the exceptions of the initiator aa-tRNA that is verified and delivered by translation initiation factors, and selenocysteinyl-tRNA that is verified and delivered by SelB. 

The second major group of aa-tRNAs is composed by misacylated translation substrates. A part is due to mistakes by the synthetases. Because elongation factor EF-Tu verifies aa-tRNAs before delivery to the ribosomes, and due to rapid editing by synthetases these errors are low: in most cases once in 10^6^ events or less [78, 79]. aaRSs have special editing domains, which are located at a distant position from the synthetic domain, to decouple amino acids from misacylated tRNAs. It has been suggested that amino acid selection of the aaRSs depends on a double sieve mechanism, in which the substrate selection at the editing site is the inverse of the substrate selection at the synthetic site. For example, during coupling at the synthetic site, the amino acids larger than the cognate will be rejected. Then subsequent translocation to the editing site takes place where amino acids smaller than the cognate will be removed [80]. Unfortunately, this model is not complete as the editing site of some aaRSs can still edit on the basis of substrate selection present at the synthetic site. In a more recent model for class I aaRSs, it is proposed that the resting state of an aaRSs has the CCA of a bound tRNA at the editing site. When the intermediate aminoacyl-adenylate is formed in the synthetic site, the CCA of the tRNA is translocated to the synthetic site, allowing aminoacyl transfer from the adenylate to the CCA. After that the aminoacylated-CCA is translocated back to the editing site, allowing inspection, and subsequent hydrolysis or release of the aa-tRNA. This model uses two translocation actions providing the opportunity for kinetic proofreading (discussed later) [78]. Besides the editing domains available in aaRSs themselves, free-standing editing proteins, homologs to aaRSs that lack the acylation domain, also exists in all three domains [81–83]. 

In addition to accidentally misacylated tRNAs, there is also a group of aa-tRNAs that is deliberately misacylated by aminoacyl-tRNA synthetases, and are subjected to pretranslational amino acid modification. In a large number of Archaea, for example, *Methanothermobacter thermautotrophicus* [84], and Bacteria glutamate and aspartate are coupled to tRNA^Gln^ and tRNA^Asn^, respectively, by a nondiscriminating aaRS, and then converted by a tRNA amidotransferase into Gln-tRNA^Gln^ and Asn-tRNA^Asn^. Other deliberate mis-acylation pathways include cysteinyl-tRNA^Cys^ (via *O*-phosphoseryl-tRNA^Cys^) in methanogenic Archaea [85], and selenocysteinyl-tRNA^Sec^ (via seryl-tRNA^Sec^) [86] (reviewed in [77]).

### 4.3. Translation

Polymerization of amino acids is catalyzed by the ribosome, a large ribonucleoprotein complex that consists of 3-4 ribosomal RNAs and a large number of ribosomal proteins [87]. Archaeal translation is initiated by recognition of the small ribosomal subunit (30S) of an initiation codon, and the formation of the initiation complex, which includes the initiation factors, the initiator tRNA (Met-tRNA^Met^) and mRNA. When the initiation complex is formed, the large subunit (50S) joins and the monomeric 70S ribosome is formed. Several mechanisms are known for initiation site recognition. Best known for prokaryotes is the mechanism that is associated with a Shine-Dalgarno (SD) motif that is recognized by the anti-SD motif on the 16S rRNA of the 30S. Although it is best known, it is not primarily used by all Bacteria or Archaea. *Sulfolobus* and *Pyrobaculum,* for example, use the SD mechanism only on distal cistrons of polycistronic transcripts, and not for the first cistron [88, 89]; in addition, *Haloarchaea,* hardly make use of this mechanism at all [90]. In Eukaryotes, that are devoid of the SD mechanism, the 40S cannot interact directly with mRNA, but needs mediation by the 5′-cap binding complex eIF4F. After binding of mRNA, it scans the RNA for an initiation codon by moving in the 3′ direction. Once located, the 60S joins the complex, the initiation factors leave, and elongation can start [91]. Less frequently, Eukaryotes use an IRES-dependent recognition mechanism, in which the complex IRES structures, that are located in the 5′-UTR, are recognized by IRES-binding transacting factors that are involved in recruitment of the small subunit [92]. All three domains of life also contain leaderless mRNAs, transcripts that start with 5′-terminal initiation codons, and that can be efficiently translated by all ribosomes regardless of the source [93, 94]. While leaderless transcripts are rare in Bacteria and Eukaryotes, they are abundant in many Archaeal species, being the primary mechanisms for monocistronic mRNAs and opening cistrons [88–90, 95]. It is thought that these leaderless transcripts are relics of primitive translation systems [93]. Recently, a novel mechanism has been identified in *Haloarchaea,* and although the exact molecular details are unknown, it has been demonstrated to act on transcripts that do not contain SD nor IRES motifs, however the efficiency of their translation depends on the 5′-UTR sequence involved [94].

On the basis of structural and chemical similarities between the homologous systems, translation elongation in Archaea is most likely very similar to that in Bacteria and Eukaryotes. Bacterial translation elongation occurs as follows. First, a ternary complex, which consist of an aminoacyl-tRNA, elongation factor EF-Tu (eEF1A/aEF1*α* in Eukaryotes/Archaea), and GTP is delivered to the Aminoacyl (A)-site. This complex reacts with the peptidyl-tRNA harboring the Peptidyl (P)-site. During this reaction, that is discussed below in more detail, the peptidyl is transferred to the aminoacyl-tRNA, elongating the nascent chain by one amino acid. Third the peptidyl-tRNA in the A-site and the deacylated tRNA in the P-site move one position to the P and the Exit (E)-site respectively, leaving the A-site empty and ready for a new round. Energy for this translocation, in which also the accompanying mRNA moves accordingly, is delivered by GTP hydrolysis by EF-G (eEF2/aEF2 in Eukaryotes and Archaea). Accuracy of the ribosome depends on (i) kinetic proofreading, (ii) induced fit, and (iii) postpeptidyl transfer quality control that will be discussed in more detail in the next paragraphs (reviewed in [14]).

### 4.4. tRNA Selection by Kinetic Proofreading and Induced Fit

Kinetic proofreading is a mechanism that allows discrimination between small energetic differences with low error rates by repeated usage of those differences in distinct separate steps and by coupling them to high-energy intermediates. The error rate drops exponentially proportional to the number of repetitions [96, 97]. During translation elongation, the energetic difference between the codon and anticodon is measured first during the encounter between ribosome and the ternary complex (initial selection), and then again after hydrolysis of GTP, which is irreversible, when the ribosome associates with either the ternary complex (with GDP instead of GTP), or the free aminoacyl-tRNA when EF-Tu is dissociated (proofreading) [14]. 

Recent models based on bacteria show that decoding is composed out of seven steps [98]. (1)  *Initial binding*: the exceptionally fast codon-independent interaction of the ternary complex to the ribosome is determined by EF-Tu and the ribosome, probably with a key role for the L7/L12 stalk. (2)  *Codon recognition*: the formation of a complementary codon-anticodon at the decoding centre, what is reflected by a correct (presumably Watson-Crick) geometry, induces conformational changes in the 16S rRNA, while near-cognate geometry induces a different structural change that leads to an almost 1000-fold higher dissociation rate, although recognition rates remain almost similar. (3)  *GTPase activation*: the GTP hydrolysis rate is increased by binding of cognate tRNAs compared to near-cognate binding. The local 16S rRNA conformational changes upon cognate binding (step (2)) lead to a closed conformation of the 30S ribosomal subunit. This conformational signal is communicated to the 50S ribosomal subunit and affects EF-Tu GTP hydrolysis. Near-cognate binding induces a different structural change in the decoding centre what most probably does not lead to the closed conformation of the 30S subunit, and thereby does not affect EF-Tu GTP hydrolysis. Slowing down hydrolysis increases discrimination capacity, however at the cost of velocity. (4)  *GTP hydrolysis*: the rate of GTP hydrolysis by EF-Tu depends on the activation state of EF-Tu. (5)  *Conformational change of EF-Tu*: EF-Tu changes from the GTP-form to the GDP form. This conformational change is limited by the rate of inorganic phosphate release. EF-Tu releases the aa-tRNA probably during the transition. (6)  *Accommodation or rejection*: after release, the 3′ end of the aa-tRNA has to move almost 70 Å from its binding site on EF-Tu to the Peptidyl Transferase Centre (PTC), while the codon-anticodon interaction should remain intact. Accommodation in the PTC of cognate aa-tRNA is rapid and efficient, in contrast to near-cognate aa-tRNA that is mostly rejected because of the low stability of binding and lower rate for accommodation. (7)  *Peptidyl-transfer*: the Peptidyl chain is transferred from the aa-tRNA on the P-site to the aa-tRNA on the A-site, elongating the nascent peptide with one amino acid. Initial selection occurs in steps (1) to (3), while proofreading occurs in step (6).

The ribosome not only uses kinetic proofreading to improve its selectivity it also uses an additional principle to further improve it: induced-fit. Induced-fit is a principle in which the correct substrate induces a conformational change leading to an acceleration of the desired process, while an incorrect substrate has the opposite effect. During decoding a correct codon-anticodon interaction accelerates both GTPase activation and accommodation steps, while a noncorrect near-cognate interaction inhibits both steps, leading to rejection [98]. It was reported that near-cognate tRNAs showed an increase in GTP consumption relative to the amount of amino acids incorporated, while other noncognate tRNAs did not. This suggests that noncognate tRNAs are already rejected during the second step whereas near-cognate are expelled during the fifth step after GTP hydrolysis, showing the importance of kinetic proofreading and induced fit for reliable discrimination between cognate and near-cognate tRNAs [99].

### 4.5. Postpeptidyl Transfer Quality Control

The molecular characteristics of Postpeptidyl transfer quality control (PPQC) have only recently been discovered in detail using bacterial in vitro systems [100], but it is likely to occur also in Eukaryotes and Archaea. Like proofreading in nucleotide polymerization, the ribosome senses mismatching after the polymerization reaction (peptidyl transfer in this case). However, where in replication and transcription exonucleases could erase the mistake, the ribosome should take more drastic actions to undo translation errors: abortive termination of the nascent peptide chain. Trigger for PPQC is mismatching between tRNA and template at the P-site of the ribosome. A mismatch at the P-site increases selection of noncognate tRNA at the A-site dramatically. After peptidyl transfer and subsequent translocation, the nascent chain contains two wrong subsequent amino acids, and both E-site as P-site harbour a mismatching tRNA. Mismatching at both E- and P-site leads to strongly stimulated release of the nascent peptide chain, increasing the rate constants for release in a range comparable to tRNA selection due to increased binding of Release Factors [100].

### 4.6. Termination

Translation terminates when a stop-codon reaches the A-site. Unlike other codons a stop-codon is recognized by proteins that mimic tRNAs: class-1 release factors (RF1s). These factors induce hydrolysis of peptidyl-tRNA, disconnecting the nascent chain from the tRNA. While Bacteria use two release factors (RF1 and RF2) that recognize different stop-codon pairs (UAA/UAG and UAA/UGA, resp.), most Archaea and Eukaryotes have a single one (aRF1 and eRF1, resp.) that recognizes all three stop-codons. Results from experiments with genuine archaeal release factors and archaeal/eukaryotic chimeras in eukaryotic in vitro translation systems suggest similar mechanisms for both [101]. An interesting variant on this theme is found in pyrrolysine-utilizing Archaea. Pyrrolysine (Pyl), the 22nd amino acid, is only found in some archaeal species belonging to the Methanosarcinaceae and two Bacteria (*Desulfitobacterium hafniense* and an uncultured *δ* *-proteobacteria) *[102]. It is encoded by the amber stop codon (UAG). Pyl-tRNA^Pyl^ is normally recognized by EF-Tu, implicating normal incorporation during elongation [103]. *Methanosarcina barkeri* contains two RFs of which only one appears to be active in termination: it was found that aRF1-1 (at least when combined as a archaeal/eukaryotic chimera) had a lower release efficiency for the UAG codon than for UAA or UGA. Comparative genomics also showed that pyrrolysine-utilizing Archaea avoid UAG as a stop codon. This suggests that in these Archaea the genetic code is changed to incorporate Pyrrolysine instead of termination [101]. Reassigning stop codons is not restricted to Archaea: the Eukaryotic ciliates *Tetrahymena thermophila* and *Euplotes aediculatus* reassigned stop codons, UAG and UAA to glutamine, and UGA to cysteine, respectively, and changed specificity of their eRF1s accordingly [104, 105]. More prominent and present in all domains of life is selenocysteine (Sec), the 21st amino acid. In Archaea selenoproteins are found in *Methanococcus, Methanocaldococcus*, and *Methanopyrus* species [106]. Sec is encoded by the opal stop codon (UGA). However in contrast to Pyr incorporation, Sec incorporation needs a special elongation factor (SelB) that via an extended domain recognizes an mRNA hairpin loop downstream of a UGA codon (the selenocysteine insertion element or SECIS) [107]. Binding of Sec-tRNA^Sec^-SelB-GTP to this structure leads to insertion of Sec at in-frame UGA codons. In contrast to Bacteria, the SECIS element is located outside of the coding region in Archaea and Eukaryotes, while the archaeal and eukaryotic SelB contain considerably shorter extensions. To overcome the distance, Eukaryotes evolved an additional adapter protein (SBP2). Additionally, it was found that the ribosomal protein L30 binds SECIS elements and influences Sec insertion. Although a similar mechanism is proposed for Archaea the adapter protein is not found [86].

### 4.7. mRNA Surveillance

In Eukaryotes mRNA quality control processes exist that act during translation to ensure the quality of the transcripts. These processes, called mRNA surveillance, are dependent on the eukaryotic release factors eRF1 and eRF3 and their paralogs Dom34 (synonym Pelota), and Hbs1 and Ski7, respectively. Three mRNA surveillance pathways are known in Eukaryotes. (i) Nonsense Mediated Decay (NMD): when premature stop codons are encountered, (ii) No-go Decay (NGD): to release stalled ribosomes, and (iii) Non-stop Decay (NSD): to rescue ribosomes that have read through a stop codon. NMD and NSD are restricted to Eukaryotes: as eRF3 and other components of the NMD system are missing in Archaea. Ski7, necessary for NSD, is even only present in the *Saccharomycetales,* although recent findings suggest that Hbs1 could take over what could mean that NSD is more widespread in Eukaryotes. In contrast, Dom34, necessary for No-go Decay is also found in Archaea. This suggests that NGD might be functionally present, although Hbs1p and eRF3 are missing in Archaea [108]. In Eukaryotes a ternary complex Dom34-Hbs1-GTP is formed, similar to the formation of the eRF1-eRF3-GTP complex used in eukaryotic translation termination. This Dom34-Hbs1-GTP ternary complex is able to recognize stalled ribosomes, leading to endonucleolytic cleavage of mRNA by Dom34 [109, 110]. In Archaea, aRF1 is able to terminate translation without the help of a RF3 ortholog, what could imply that the paralogous Dom34 might be able to perform NGD without the help of an Hbs1p ortholog [108].

 To rescue stalled ribosomes, Bacteria have a system that uses an intermediate between tRNA and mRNA: tmRNA, an RNA molecule with a tertiary structure similar to tRNAs, but with an extended anticodon loop that contains an mRNA-like ORF. If the ribosome stalls, because a transcript is finished without a proper termination, tmRNA in concert with SmpB and EF-Tu binds to the empty A-site of the stalled ribosome. After translocation to the P-site, the mRNA-like ORF located in the anticodon loop of the tmRNA takes over the role of messenger, and encodes for a degradation tag and ends with a proper stop-codon. After release the nascent peptide is thus tagged for degradation, and the ribosomal subunits are released again. This system seems to be restricted to Bacteria as tmRNA genes have not been identified in Eukaryotes, with the small exception of a few eubacterial-like organelles, or Archaea [111]. Interestingly, in investigations of archaeal protein degradation in *Methanococcus jannaschii,* green fluorescent proteins tagged with a ssrA-extension were used. The ssrA extension is the 11 amino acid degradation tag encoded on the tmRNA, which gene was designated ssrA. Tagged proteins showed a rapid unfolding and degradation while untagged proteins did not [112].

## 5. Turnover of RNA and Proteins

### 5.1. RNA Decay

Beside above mentioned mRNA surveillance during translation, more general systems are involved in RNA turnover. Main component in these mechanisms in Archaea is the exosome, a protein complex that includes Rrp41 and Rrp42, a homolog of RNasePH, a bacterial phosphorolytic nuclease, and Rrp4 and Csl4, containing KH/S1 RNA-binding domains. The archaeal exosome is responsible for 3′→5′ degradation of RNA, as well as for 3′ polyadenylation. This complex is similar to the bacterial PNPase and the eukaryotic exosome. All three have a double-doughnut-like structure with a central hole with a core ring of six RNasePH-type subunits. The narrow neck of the archaeal structure only allows single-stranded RNA devoid of secondary structures, suggesting a regulatory role for cofactors, as observed in Eukaryotes [113–115].

Polyadenylation occurs mainly on fragmented molecules as part of an RNA decay pathway in Bacteria, Archaea, and eukaryotic cell organelles, and recently has been described for nuclear genes from Eukaryotes as well. Although, in contrast, in Eukaryotes poly(A) tails are also added to mature 3′ ends of most nuclear encoded, full-length, mRNAs for proper translation initiation, and mRNA stability. The general scheme of RNA 3′→5′ degradation in prokaryotes is as follows: (1) removal of the 5′ pyrophosphate, (2) endonucleolytic cleavage of the transcript, (3) poly-adenylation of cleavage products, and (4) rapid exonucleolytic degradation of polyadenylated products. In *Sulfolobus*, the exosome is able to generate a heteromeric poly(A)-rich tail and use NDPs as a substrate. It has been suggested that polyadenylation is used to overcome secondary RNA structures that otherwise cannot pass the exosome neck. Interestingly, halophilic Archaea, together with several methanogenic Archaea, like *Haloferax*, and *Methanococcus*, are the only known organisms that lack polyadenylation, and do not contain an exosome or PNPase. In these organisms poly(A)-independent RNA degradation is performed by RNase R [116–119].

Eukaryotes also use another pathway for mRNA degradation that involves 5′→3′ exonucleases, like Xrn1p. Eukaryotic transcripts are protected against this rapid form of decay by a 5′ cap. To prevent transcripts from being decapped unintentionally, they are protected by the eukaryotic translation initiation factor eIF4E [120]. In Archaea, mRNAs are similarly protected from 5′→3′ decay by binding of the *γ*-subunit of the archaeal translation initiation factor aIF2 to the 5′ end. The similarities between both systems suggests that 5′→3′ decay is common to all domains of life [121]. Additionally, this protection offers a mechanism to discriminate between new versus already translated transcripts. After translation aIF2 is removed from the mRNA, making the mRNA vulnerable to 5′→3′ decay as soon as translation is terminated. Interestingly, a tight coupling beyond the use of an initiation factor to protect mRNA, exists between transcription and translation in Archaea, as it has been found that multiple rounds of translation already start before transcription is finished [122]. This tight interplay might have to be extended to mRNA degradation as well, what would provide Archaea with a very efficient and short information processing pipeline.

### 5.2. The Protein Waste Bin

The 20S proteasome, present in Eukaryotes, Archaea, and actinobacteria, is a barrel-shaped complex that consists of four heptameric rings of *α*- and *β*-type subunits in an *α*7*β*7*β*7*α*7 configuration. Other Bacteria use the simpler HslV protease, that is structurally related to the *β*-type subunits of the 20S proteasomes. The function of the proteasome is to breakdown proteins into short peptides that in turn can be further degraded to amino acids by peptidases to be recycled in protein synthesis or in metabolism. The proteasome is therefore an essential component for protein turnover and to maintain protein quality control by degrading misfolded and denatured proteins [123, 124]. 

The protease domains of *β*-type subunits are located on the inside of the barrel. This creates a tightly regulated environment, to circumvent uncontrolled protein breakdown. In Eukaryotes, the 20S proteasome can be capped by 19S regulatory particles (a combination of a Rpt and Rpn proteins forming a base and a lit), on one side (26S proteasome), or on both sides (30S proteasome). These caps play a role in recognition and degradation of polyubiquitin tagged substrates. Archaea encode orthologs of Rpt called Proteasome-activating Nucleotidase (PAN). PAN is able to unfold proteins in a ATP-dependent manner, can open the axial gate of the 20S proteasome, and subsequently translocates the substrate into the 20S core. Interestingly, as mentioned earlier, the archaeal PAN is able to distinguish between a ssrA-tagged or untagged green fluorescent protein, which suggests a role for ssrA-tagging in peptide degradation in Archaea, although a tmRNA system responsible for ssrA-tagging has not been identified [112, 123, 124]. 

In Eukaryotes, ubiquitin and ubiquitin-like proteins, small stable proteins that contain a *β*-grasp fold and that can be attached to a wide variety of other proteins, play an important role in targeted degradation by the proteasome. Ubiquitylation is also used in a number of other nonproteolytic mechanisms like endocytosis, intracellular trafficking, chromatin-mediated regulation of transcription, and DNA repair. Discrimination between those target processes is thought to be dependent on the differences in ubiquitin chains [125]. Ubiquitin-targeted degradation is used for quality control in Eukaryotes. Misfolded proteins in the cytosol are recognized by chaperones, because of their toxic hydrophobic surfaces. These chaperones recruit ubiquitylation enzymes (e.g., CHIP), that attach a polyubiquitin chain to the misfolded protein after which it is degraded or refolded [126]. In the lumen of the endoplasmatic reticulum, N-linked oligosaccharides indicate the folding stage, but also appear to keep track of the time a polypeptide resides within the lumen. If misfolding occurs and the polypeptide is trapped within the lumen, they are directed to a ubiquitin ligase and targeted for destruction [127].

Although ubiquitin-like tagging was long thought to be restricted to Eukaryotes, an ubiquitin-like tagging system was recently revealed in *Haloferax volcanii* that is able to tag proteins with small archaeal modifier proteins (SAMPs). SAMPs are small proteins that contain a *β*-grasp fold and a C-terminal diglycine motif similar to ubiquitin, and are widespread among the Archaea. It was shown that SAMPs are coupled to a wide range of proteins. SAMP1 appears to target proteins for destruction by the proteasome [128]. Alternative signalling objectives might also be present, as SAMP2 was also found to be coupled to a wide range of proteins like SAMP1, but showed decreasing levels in proteasomal mutant strains [128]. It seems to be likely that systems similar to eukaryotic ubiquitin-targeted systems, like targeted destruction, are also present in the archaeal domain. Opening up a potential role for the proteasome in regulation of protein levels, quality control against misfolded proteins, and recycling of nonfunctional polypeptides in archaeal cells.

## 6. Concluding Remarks

It is obvious that a certain level of fidelity of genetic information processing is of major importance to the cell, in order to maintain the delicate balance between accuracy on the one hand and velocity on the other. At the moment, a rather complete picture is emerging for the three main polymerization reactions related to genetic information processing in living cells in general. More and more is known about the mechanisms and the role of factors that contribute to fidelity in these systems. To some extent these crucial cellular processes have successfully been studied in selected Archaea. Despite this progress, however, it is obvious that insight in fidelity-related mechanisms in Archaea is still relatively scarce. For that reason, extrapolations on the basis of analogous systems of Bacteria and Eukaryotes have been used in this overview to bridge the gaps in our understanding of the archaeal counterparts. Although we think that most of the described processes work similarly in Archaea, we cannot rule out that such generalisations may in some instances turn out to be an oversimplification of the actual situation. As many Archaea thrive in extreme environments, it will be very interesting to learn how fidelity mechanisms of these extremophilic organisms are adapted to overcome these harsh conditions. It is therefore anticipated that Archaea will continue to play an important role in future research to elucidate details on the intriguing systems that control the fidelity of information processing.

## Figures and Tables

**Figure 1:**
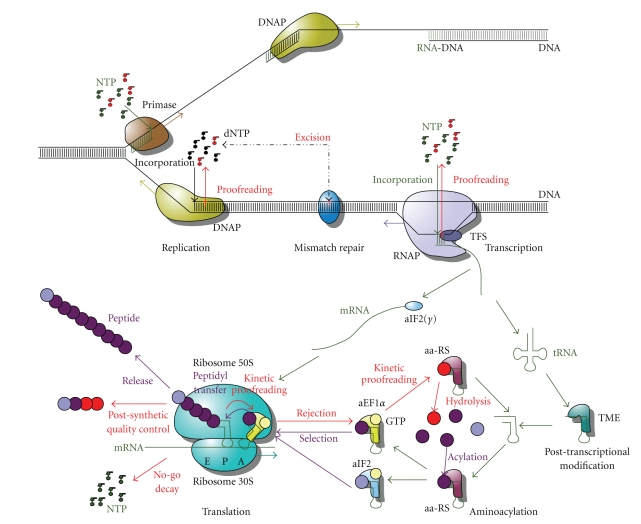
Overview of the processes involved in genetic Information Processing in Archaea (TFS: Transcription Factor S; TME: tRNA modifying enzymes; aa-RS: aminoacyl-tRNA synthetase; aIF2(*γ*): archaeal Initiation Factor 2(*γ*); aEF1*α*: archaeal Elongation Factor 1*α*).
